# Muscone abrogates breast cancer progression through tumor angiogenic suppression via VEGF/PI3K/Akt/MAPK signaling pathways

**DOI:** 10.1186/s12935-024-03401-6

**Published:** 2024-06-20

**Authors:** Danhong Wang, Xiaozhen Liu, Weimin Hong, Tianzheng Xiao, Yadan Xu, Xiang Fang, Hongchao Tang, Qinghui Zheng, Xuli Meng

**Affiliations:** 1 College of Pharmacy, Zhejiang University of Technology, Hangzhou, 310014 Zhejiang China; 2 Zhejiang Provincial People’s Hospital, Affiliated People’s Hospital, Hangzhou Medical College, Hangzhou, 310053 Zhejiang China; 3 Department of Breast Surgery, General Surgery, Cancer Center, Zhejiang Provincial People’s Hospital, Affiliated People’s Hospital, Hangzhou Medical College, Hangzhou, 310014 Zhejiang China; 4 Key Laboratory for Diagnosis and Treatment of Upper Limb Edema and Stasis of Breast Cancer, Hangzhou, 310014 Zhejiang China; 5 College of Clinical Medicine, Jinzhou Medical University, Jinzhou, 121001 Liaoning China

**Keywords:** Muscone, Breast cancer, Tumor angiogenesis, VEGF/, PI3K/, Akt/, MAPK signaling pathway axis

## Abstract

**Background:**

Angiogenesis strongly reflects poor breast cancer outcome and an important contributor to breast cancer (BC) metastasis; therefore, anti-angiogenic intervention is a potential tool for cancer treatment. However, currently used antibodies against vascular endothelial growth factor A (VEGFA) or inhibitors that target the VEGFA receptor are not effective due to weak penetration and low efficiency. Herein, we assessed the anti-BC angiogenic role of muscone, a natural bioactive musk constituent, and explored possible anti-cancer mechanisms of this compound.

**Methods:**

CCK-8, EdU, scratch and Transwell assessments were employed to detect the muscone-mediated regulation of breast cancer (BC) and human umbilical vein endothelial cells (HUVECs) proliferation and migration. Tube formation, matrigel plug assay and zebrafish assay were employed for assessment of regulation of tumor angiogenesis by muscone. In vivo xenograft mouse model was constructed to compare microvessel density (MVD), vascular leakage, vascular maturation and function in muscone-treated or untreated mice. RNA sequencing was performed for gene screening, and Western blot verified the effect of the VEGFA-VEGFR2 pathway on BC angiogenic inhibition by muscone.

**Results:**

Based on our findings, muscone suppressed BC progression via tumor angiogenic inhibition in cellular and animal models. Functionally, muscone inhibited BC cell proliferation and migration as well as tumor cell-conditioned medium-based endothelial cell proliferation and migration. Muscone exhibited a strong suppressive influence on tumor vasculature in cellular and animal models. It abrogated tumor cell growth in a xenograft BC mouse model and minimized tumor microvessel density and hypoxia, and increased vascular wall cell coverage and perfusion. Regarding the mechanism of action, we found that muscone suppressed phosphorylation of members of the VEGF/PI3K/Akt/MAPK axis, and it worked synergistically with a VEGFR2 inhibitor, an Akt inhibitor, and a MAPK inhibitor to further inhibit tube formation.

**Conclusion:**

Overall, our results demonstrate that muscone may proficiently suppress tumor angiogenesis via modulation of the VEGF/PI3K/Akt/MAPK axis, facilitating its candidacy as a natural small molecule drug for BC treatment.

**Supplementary Information:**

The online version contains supplementary material available at 10.1186/s12935-024-03401-6.

## Introduction

In 2023, breast cancer (BC) made up 31% of all new cancer diagnosis among women, rendering it as the leading category of cancer in women [1]. Currently, there is no cure for advanced BC with distant organ metastases, and available management approaches include both local (surgery and radiotherapy) and systemic therapies, such as, chemotherapy, endocrine therapy for hormone receptor-positive disease, anti-HER2 therapy for HER2-positive disease, bone stabilizing agents, poly (ADP-ribose) polymerase inhibitors for BRCA mutation carriers and immunotherapy [2]. Although systemic therapies significantly prolong survival of BC patients, they are associated with serious side effects.

Aberrant angiogenesis is a well-known hallmark of BC. Blood vessels growing within the tumor help to provide nutrients for tumor development, and BC angiogenesis is a strong indicator of worse outcome and a major risk factor for BC metastasis [3, 4]. Accordingly, anti-angiogenic therapy is a crucial approach in BC and many other solid tumor interventions [5]. More than a dozen anti-angiogenic drugs received FDA approval for management of a number of cancer types [6]. Nonetheless, reduced efficacy, toxic complications, unsatisfactory pharmacokinetic effects and increased expenses restrict their widespread clinical use [6–8]. Therefore, the development of natural anti-angiogenic drugs with improved therapeutic efficacy and low toxicity is crucial in the fight against BC [9].

Musk, the dried release from the male musk deer (*Moschus berezovskii*) ventral glands [10], is often applied in traditional Chinese medicine for disease management, particularly, stroke, coma, neurasthenia, convulsions, heart disease, and ulcerative sores [11–13]. Muscone is the primary bioactive constituent of musk, and it has numerous biological influences, such as anti-inflammatory activity [14], cardiovascular and cerebrovascular protective activity, and neuroprotective activity [15, 16]. Muscone also has multiple anticancer activities, namely activation of the mitochondria-mediated pro-apoptotic pathway in Ehrlich ascites tumor cells [17]. It also promotes HepG3 cell apoptosis via modulating the PERK/ATF2/DDIT4 axis and to inhibit the hepatocellular carcinoma progression by inducing autophagy via a SESN2-based AMPK network activation [18]. Despite clear impact of muscone on cancer cell biology, potential roles for muscone in BC management is yet unexplored.

Herein, we examined the potential impact of muscone in BC. The results of cellular and animal experiments led us to focus on the antiangiogenic role of this molecule, and mechanistic investigations showed an interaction of muscone with VEGF/PI3K/Akt/MAPK signaling pathways. Both cellular and animal experiments further revealed that muscone inhibits tumor angiogenesis promotes tumor vascular normalization through its effects on these pathways, thereby inhibiting BC progression.

## Materials and methods

### Cell culture and tumor cell-conditioned medium (TCM) preparation

We obtained the following cell lines: Human BC MDA-MB-231 and BT-549, human breast epithelial MCF-10A, and human umbilical vascular endothelial cells (HUVECs) from the American Type Culture Collection (ATCC, Manassas, VA, USA). MDA-MB-231 cells were grown in RPMI-1640 containing fetal bovine serum (FBS; 10%) and penicillin and streptomycin (PS; 1%); HUVECs and BT-549 cells were placed in DMEM with FBS (10%) and PS (1%); and MCF-10A cells in DMEM/F12 medium with horse serum (5%), epidermal growth factor (20 ng/mL), hydrocortisone (0.5 µg/mL), human insulin (10 µg/mL), non-essential amino acids (1%), and PS (1%). Cells were incubated at 37 °C and with 5% CO_2_.

To prepare tumor cell-conditioned medium (TCM), MDA-MB-231 and BT-549 cells were plated in 6-well plates and cultured with 2 mL of DMEM. Following overnight (ON) incubation, cells were exposed to several muscone concentrations (0, 7.5, 15, 30, 60, or 120 µM) (Topscience, Shanghai, China) for 48 h. TCM was then collected, prior to a 10-min centrifugation at 2000 × g for cellular debris removal, and storage at −80 °C.

### Cell survivability assays

The Cell Counting Kit-8 (CCK-8) (Topscience, Shanghai, China) was employed for determination of the muscone-based regulation of cell survivability. Approximately 4 × 10^3^ cells were introduced to individual wells of 96-well plates, and plates underwent a 24 h-incubation at 37 °C and 5% CO_2_ humid chamber [19–21]. Cells were exposed to several muscone concentrations followed by 24, 48, or 72 h incubation. Following medium removal, 100 μL fresh medium with 10% CCK-8 reagent was introduced to wells, prior to a 2-h maintenance at 37 °C. Optical density was measured at 450 nm via a microplate reader. GraphPad Prism 8.0 (GraphPad Inc., La Jolla, San Diego, CA, USA) was employed for dose–response curves, and half maximal inhibitory concentrations (IC_50_) were computed via nonlinear regression analysis.

### Wound healing and transwell migratory assessment

Cells were plated in 6-well plates and grown for 24 h at 37 °C until 80–90% confluency, subsequently, the cellular monolayer was scratched with a sterile 200 µL pipette tip followed by a PBS rinse to eliminate nonadherent cells. Scratched cells were maintained with or without muscone (30 or 60 µM) in serum-free medium (SFM) for 24 h.

Cell migration assays employed transwell chambers (Corning, NY, USA) with a filter with 8 µm pore size. Cells (4 × 10^4^ in 100 µL SFM) were introduced to the top compartment of a 24-well transwell plate, and DMEM with 10% FBS (800 µL) was introduced to the bottom compartment. Following a 24-h incubation, cells that migrated underwent a 40-min fixation in 4% paraformaldehyde (PFA) and 40-min staining in 0.1% crystal violet. The plates were subsequently PBS rinsed, and cells that remained in the top compartment were removed using cotton swabs. The plates and the migrated cells underwent image capture via an EVOS M7000 microscope (Thermo Fisher Scientific, USA), and images were analyzed with Fiji imaging software.

### EdU incorporation assay

In all, 4 × 10^4^ cells were plated per well of a 24-well plate. Following a 48-h incubation, the cells underwent a 2-h treatment with EdU (Beyotime, Shanghai, China) at 37 °C, prior to fixation in 4% PFA, a PBS rinse, a 15-min permeabilization in 0.5% Triton X-100 at room temperature (RT), and incubation in a click additive solution in the dark, followed by Hoechst 33342 staining, and image capture with an EVOS M7000 microscope (Thermo Fisher Scientific, USA).

### Tube formation assay

Frozen Matrigel (Corning, USA) was ON thawed at 4 °C, then introduced to 96-well plates (50 µL/well), followed by a 30-min incubation at 37 °C for solidification. Subsequently, 2 × 10^4^ cells in 100 µL TCM with varying muscone concentrations (0–60 µM) were plated on the Matrigel. Approximately 4 h later, the HUVEC tubular structures were observed and image capture was performed via an inverted light microscope (ZEISS Axio Observer 3, Germany), and tube lengths were analyzed using Fiji imaging software.

### RNA-sequencing and functional enrichment analyses

Total RNA was isolated from vehicle (0.1% DMSO)- or 60 μM muscone-treated MDA-MB-231 cells (n = 3 for both treatment groups) using TRIzol. BioNovoGene (Suzhou, China) performed all RNA-sequencing and gene expression assessments. DESeq identified differentially expressed genes (DEGs) between vehicle- and muscone-exposed cells via the following criteria: |log2FoldChange|> 1 and *P* < 0.05. GO and KEGG network assessments were conducted via R.

### Reverse transcription-quantitative PCR (qRT-PCR)

Total RNA isolation from TNBC cells employed the RNA-Quick Purification Kit (RN001, ESscience Biotech, Shanghai, China) and associated directions. Overall, 1 µg RNA was converted to cDNA via the Fast All-in-One RT Kit (RT001, ESscience Biotech, Shanghai, China). Then, relative gene expression was computed via qRT-PCR using the 2 × SYBR Green qPCR Master Mix (B21203, Bimake, Shanghai, China) and the Applied Biosystems 7500 Real Time PCR System (Thermo Fisher Scientific, USA). All employed primers are detailed in Table S1.

### Western blotting

Total protein extraction was carried out with RIPA cell lysis buffer (Solarbio, Beijing, China) with protease and phosphatase inhibitor cocktails (Proteintech, Wuhan, China), and protein was quantified via a BCA Protein Assay Kit (Thermo Scientific). Protein separation utilized an 8% or 12.5% SDS–polyacrylamide gel electrophoresis and transfer was done to polyvinylidene difluoride membranes, which were next blocked with 5% milk, prior to an ON treatment with primary antibody at 4 °C, with subsequent 1-h treatment with corresponding secondary antibodies at RT. Protein visualization was done with an ECL kit (Applygen Technologies Inc., Beijing, China). Table S2 summarizes all employed primary antibodies.

### Enzyme-linked immunosorbent assay (ELISA)

TCM was assayed to evaluate vascular endothelial growth factor A (VEGFA) content via an ELISA kit (ABclonal Biotechnology co., Ltd., Wuhan, China) and associated directions.

### Matrigel plug evaluation

The in vivo antiangiogenic muscone action was examined using the Matrigel plug model [22]. Female BALB/c mice were separated into 5 cohorts (n = 4), prior to a subcutaneous administration of 500 µL Matrigel (Mogengel Bio, Xiamen, China) with 40 U heparin, recombinant human VEGF165 (100 ng/mL) (Novoprotein, Suzhou, China) and varying muscone concentrations (0, 30, 60, 120 µM). VEGF165-free matrigel served as the control. Subsequently, mice were sacrificed 12 d later. Following matrigel plugs harvest, they underwent ON fixation in 4% glutaraldehyde, followed by CD31 immunofluorescence (IF) staining. Microvessel imaging was done via a Leica SP5 confocal microscope, and microvessel density (MVD) quantification utilized the Fiji imaging software.

### In vivo studies in zebrafish

The transgenic zebrafish line Tg(fli-1a:EGFP), harboring the enhanced green fluorescent protein (EGFP) in endothelial cells, was generously gifted by Professor Xia, Zhejiang University. Zebrafish were maintained at 28.5 °C and a 10/14 h dark/light cycle. The night prior to emodin exposure, both male and female zebrafish were housed in a system featuring a fish mating cage with an inner mesh and divider. Embryos were harvest following natural spawning then washed in system water, prior to maintenance in E3 embryo medium (5 mM NaCl, 0.17 mM KCl, 0.33 mM CaCl_2_, and 0.33 mM MgSO_4_, pH 7.2) at 28.5 °C. After 12 h of fertilization, 0.003% 1-phenyl-2-thiourea (PTU) was introduced to minimize pigment production [23]. To examine the muscone-mediated regulation of tumor environment vasculature, approximately 3 × 10^4^ Dil-labeled (VybrantTM DiI) MDA-MB-231 cells were administered to the perioocyte suture. Muscone was resuspended in DMSO followed by dilution in fish water to achieve a 30 μM final concentration. Chemicals were replaced daily. At 3 days following fertilization, larvae underwent 0.168 mg/mL tricaine (Sigma-Aldrich, USA)-based anesthesia, and image capture was completed with an Axio Zoom.V16 for Biology microscope (ZEISS, Germany).

### In vivo xenograft mouse model

MDA-MB-231 cells (5 × 10^6^ in 100 μL PBS) were subcutaneously administered to the right armpits of 4- to 6-week-old nude female mice (SLAC, Shanghai, China). Then, 1-week later, mice were arbitrarily separated into 3 cohorts (n = 5). Over the course of the 21 d experiment, control mice were administered with normal saline via intragastric administration, once every 2 days; and mice of the reduced- and augmented-dose cohorts received muscone of 2 or 4 mg/kg via intragastric administration, respectively, once every 2 days [15, 24, 25]. Tumor volumes and nude mice weights were recorded every 2 d. Tumor volume was computed as (length × width^2^)/2. At end of treatment, mice were sacrificed, and tumors excised and weighed. All animal protocols received ethical approval from the institutional review board (Ethics Number: 20231109105746519905).

### Pimonidazole staining and perfusion assessment

Tumor hypoxia was assessed by injecting mice with Hypoxyprobe-1 (60 mg/kg, Hypoxyprobe) via intraperitoneal injection 1 h prior to sacrifice. Tumors were excised before embedding in Tissue-Tek OCT, then treated with a FITC-conjugated mouse anti-pimonidazole monoclonal antibody, as directed in associated protocols. The vessel perfusion was measured via a DyLight 488-conjugated tomato lectin (1 mg/mL, 100 µL, Vector Laboratories) via intravenous injection 30 min prior to sacrifice. Tumors were excised for additional exploration.

### Hematoxylin and eosin (H&E), immunohistochemical (IHC) and IF staining

For IF-based exploration of tissues, samples underwent fixation in 4% PFA, then a 24-h dehydration in 30% sucrose, and embedding in Tissue-Tek OCT compound. Frozen tissue blocks were sliced into 10-μm-thick sections. To conduct IF-based exploration of cells, cells underwent fixation in 4% PFA, then a 15-min permeabilization in 0.5% Triton, prior to blocking in 5% goat or donkey serum in PBS with 0.1% Tween-20 and subsequent ON incubation in VEGFA-targeting primary antibody (ABclonal, Wuhan, China) at 4 °C. We conducted several rinses, prior to a 1-h treatment with goat anti-rabbit IgG H&L (Alexa Fluor^®^ 488) antibody (Abcam, UK) at RT. Nuclear staining used DAPI (Beyotime, Shanghai, China), image capture was done with a Leica SP5 confocal microscope, and analysis with Fiji.

Paraffinized tumor sections underwent H&E staining for morphological evaluation. We also stained tumors with Ki67-targeting primary antibody (Abcam, UK) and HRP-conjugated goat anti-rabbit IgG H&L (Servicebio, Wuhan, China) for IHC assessment. Observation was done under an EVOS microscope, and positive cell quantification utilized Fiji.

### Statistical analysis

Prism (GraphPad Inc, version 8.0) was used for all data analyses. All quantitative data are provided as mean ± SEM. Inter-cohort differences were assessed using unpaired Student’s t-tests. Multi-cohort differences were assessed with one- or two-way ANOVA. *P* < 0.05 was the significance threshold.

## Results

### Muscone inhibits MDA-MB-231 and BT-549 cell proliferation and migration in vitro

**Fig. 1:**
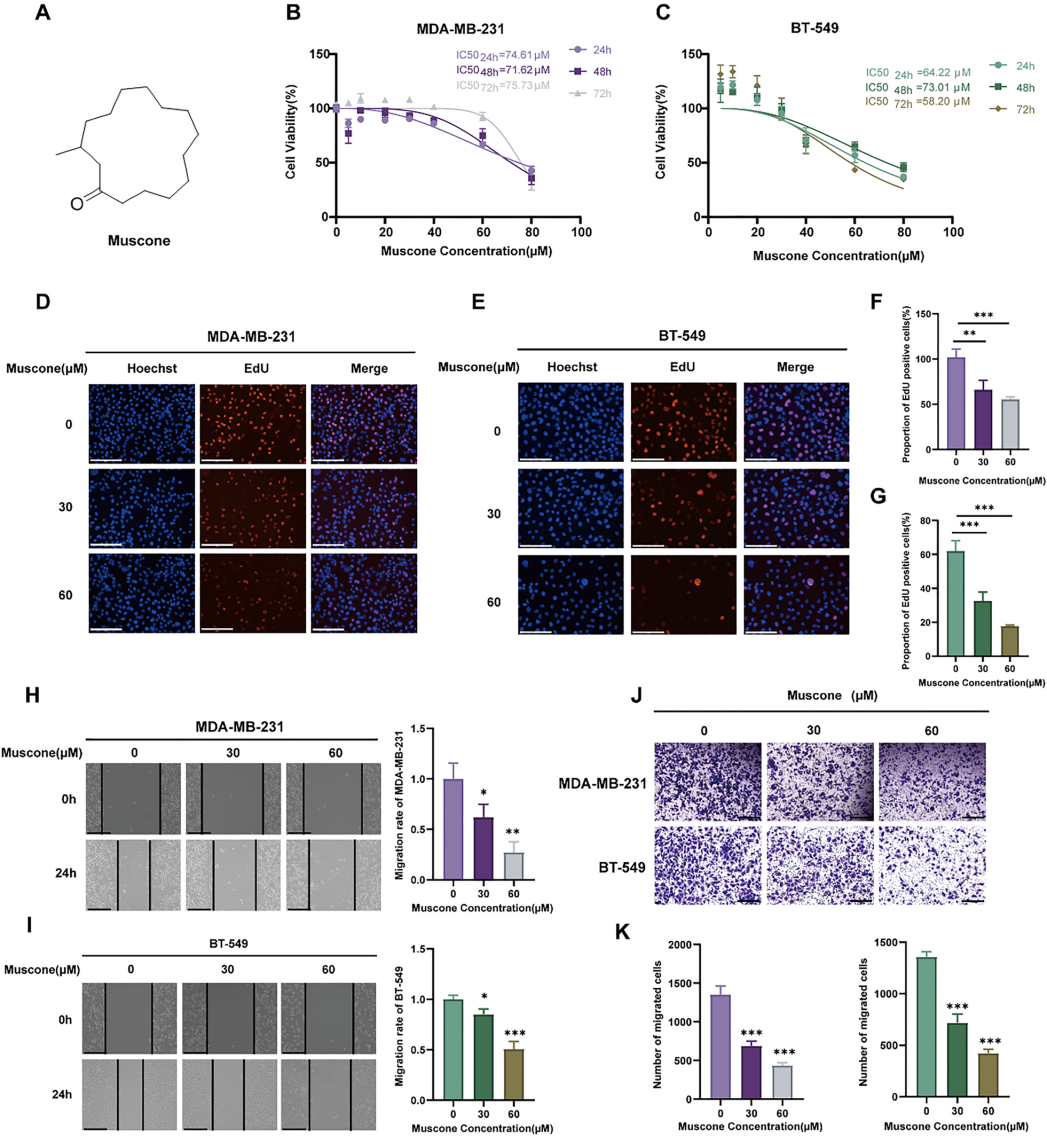
Muscone diminishes BC cell proliferation and migration in vitro. **A** The chemical muscone configuration. **B**, **C** The muscone-mediated regulation of BC cell survivability (MDA-MB-231 and BT-549) as detected via CCK-8 assessment. **D**–**G** Representative images and quantification of EdU proliferation assays performed in BC cells exposed to vehicle or specified muscone concentrations; scale bar, 500 µm. **H**, **I** Scratch wound assays of monolayers of BC cells; scale bar, 1000 µm. **J**, **K** The muscone-mediated regulation of BC cell migration, as detected by Transwell cell migration assay. Typical depiction (**J**) and quantification of migrated BC cell quantity (**K**); scale bar: 1250 µm. Data is shown in mean ± SEM. n = 3. *P < 0.05, **P < 0.01, and ***P < 0.001

We tested the effects on MDA-MB-231 and BT-549 cell survivability following treatment with varying muscone concentrations for 24, 48, and 72 h. The muscone structure is presented in Fig. 1A. We found that muscone dramatically diminished the MDA-MB-231 and BT-549 cell survivability in a concentration-reliant fashion, with IC_50_ values (48 h) of 71.62 μM in MDA-MB-231 cells and 73.01 µM in BT-549 cells (Fig. 1B, C). However, muscone showed a less potent effect on human breast epithelial MCF-10A cells (Figure S1). Muscone also decreased the cell proliferation of these cell types, as the frequency of EdU-positive cells was diminished following muscone administration (Fig. 1D, E).

Because augmented invasion and aggressive metastasis are typical characteristics of BC cells, we next tested the muscone-mediated regulation of BC cell migration.As shown in Fig. 1F, G, we revealed that muscone suppressed scratch wound closure in wounded monolayers of both MDA-MB-231 and BT-549 cells. Muscone exposure also dramatically reduced BC cell migration (Fig. 1H) in a concentration-reliant fashion. Collectively, these findings suggest that muscone produces anti-proliferative and anti-invasive influences on BC cells in vitro.

### Muscone inhibits tumor-conditioned medium-stimulated HUVECs cell proliferation and migration

**Fig. 2:**
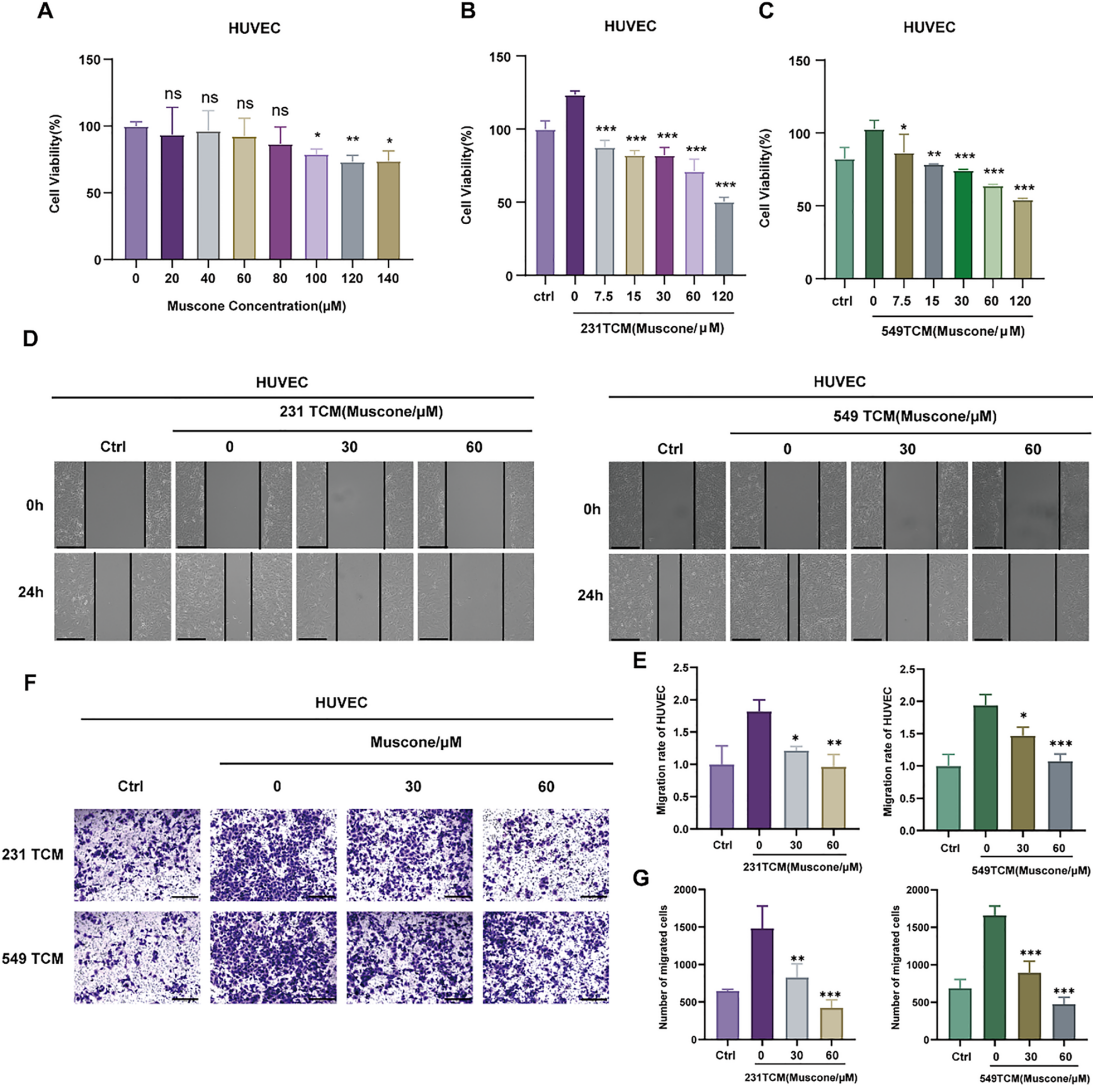
Muscone suppresses TCM-induced HUVECs proliferation and migration in cellular models. **A** The muscone-based regulation of HUVECs viability, as assessed via CCK-8 assays. **B**, **C** Influence of TCM-exposed MDA-MB-231 and BT-549 cells pretreated with muscone on HUVECs viability, as detected via CCK-8 assays. **D**, **E** Scratch wound assay of HUVEC cells treated with TCM from tumor cells pretreated with muscone; Scale bar, 1000 µm. **F**, **G** The influence of TCM from cells pretreated with muscone on HUVECs cell migration, as detected via Transwell cell migration assays. Typical representation (**F**) and quantification of migrated HUVECs quantity (**G**). Scale bar: 1250 µm. Data is shown as mean ± SEM. n = 3. *P < 0.05, **P < 0.01, and ***P < 0.001 vs TCM from untreated cells

Circulating vascular endothelial cells are typically inactive until induced to proliferate by angiogenesis factors. To assess the performance of muscone-regulated angiogenic activity, we conducted a series of assays using HUVECs. Firstly, we investigated the effect of muscone on HUVECs. As shown in Fig. 2A, little cytotoxicity was evident in muscone-treated HUVECs. We next examined the muscone-regulated how BC cell exposure to TCM influenced the behavior of HUVECs. However, TCM from muscone-pretreated tumor cells led to significant decreases in the proliferation of HUVECs (Fig. 2B, C). We also found that TCM without muscone-pretreated which called blank TCM induced the closure of wounds in monolayers of HUVECs, but TCM from muscone-pretreated tumor cells inhibited closure of wounds in monolayers of HUVECs (Fig. 2D, E), and muscone pretreatment significantly decreased the induction of HUVECs cell migration by the resulting TCM in a concentration-reliant fashion (Fig. 2F, G). Collectively, these evidences revealed that muscone treatment of tumor cells led to the production of TCM with a lower ability to induce HUVECs cell proliferation and migration.

### Muscone inhibits tumor angiogenesis in cellular and animal models

**Fig. 3:**
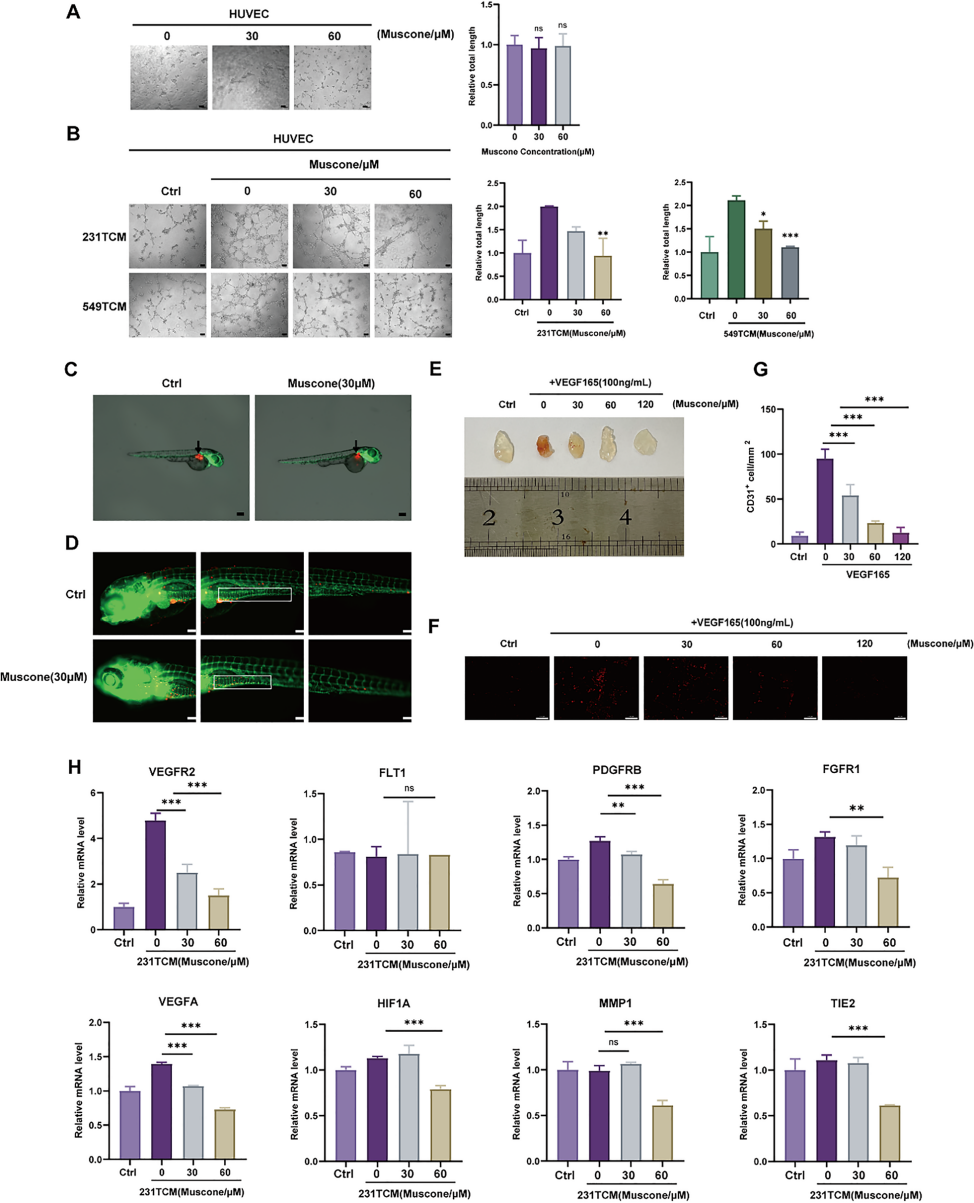
Muscone abrogates tumor angiogenesis in cellular and animal models. **A**, **B** Tube formation in vitro of HUVECs treated with muscone (**A**), or TCM from muscone-treated MDA-MB-231 cells (231TCM) or BT-549 cells (549TCM) (**B**). Tube lengths were measured with Fiji imaging software; Scale bar, 100 µm. **C** Equal numbers of Dil-labelled and incorporated MDA-MB-231 cells were administered into the zebrafish embryos perivitelline space at 48 h after fertilization. Arrows mark the administration locations. Magnification: 28.5 × . **D** Vasculature and neo-angiogenesis around the primary tumor and metastasis locations in zebrafish treated with muscone for 48 h. Boxes indicate newly formed tumor vessels. Magnification: 100 × . **E** Photographs of the Matrigel plugs from mice treated with VEGF and muscone for 12 d. **F** Representative images of immunofluorescent CD31^+^ microvessels (red) within Matrigel plugs; Scale bar, 100 µm. **G** Microvessel densities quantified as the density of CD31^+^ cells.** H** The effect of TCM from muscone-treated MDA-MB-231 cells (231TCM) on tumor angiogenesis-related gene expression in HUVECs. All data are shown as mean ± SEM, n = 3, *P < 0.05, **P < 0.01, and ***P < 0.001

To examine the muscone-mediated regulation of tumor angiogenesis in cellular models, we cultured HUVECs using TCM with MDA-MB-231 or BT-549 pre-exposed or not exposed to muscone. As depicted via tube formation assessments (Fig. 3A, B), HUVECs grown in MDA-MB-231 and BT-549 cell TCM formed prolonged tube structures relative to those grown in normal medium. Nonetheless, TCM from MDA-MB-231 and BT-549 cells pre-exposed to muscone produced HUVEC tubes with markedly shorter tubes relative to untreated TCM, however with lengths a little augmented, relative to those produced by HUVECs treated with normal medium. This series of experiments suggested that muscone inhibited TCM-stimulated angiogenesis via suppression angiogenic signal release from tumor cells.

To further explore muscone angiostatic activity in mice, we employed transgenic zebrafish (Fli1a: EGFP) to examine the muscone-mediated regulation of zebrafish tumor angiogenesis [23] at 30 µM dosage, which is known to lead to no detectable toxic effects. Following treatment of these zebrafish with muscone, we observed the level of angiogenesis around the primary tumor and metastatic locations. As shown in Fig. 3C, D, in muscone-treated zebrafish, angiogenesis around the tumor, especially as observed by the intensity of blood vessel-related signal originating from the subintestinal area, was diminished relative to the control zebrafish. Moreover, zebrafish treated with muscone had shorter cell migration distances than did the control group.

The Matrigel plug model [22] was also employed to evaluate the muscone antiangiogenic activity in vivo. As shown in Fig. 3E, plugs treated with muscone prior to implantation were more pale than were non-pretreated plugs at 12 d after implantation, indicating that fewer new vessels were formed near the plugs containing muscone. Immunofluorescent CD31-positive blood vessels within plugs similarly suggested that muscone abrogates angiogenesis in a concentration-reliant fashion (Fig. 3F, G).

To further test whether muscone can inhibit TCM-induced angiogenesis, we observed tumor angiogenesis-related gene expression in HUVECs. While TCM from untreated BC cells induced increased expression of VEGFR2, VEGFA, FGFR1, HIF-1α and PDGFRB, this effect was significantly inhibited by the muscone administration to tumor cells prior to isolation of the 231TCM (Fig. 3H) and 549TCM (Figure S2).

Together, these results revealed that muscone exerts antiangiogenic effects on BC cells in cellular and animal models.

### Muscone inhibits the VEGF axis in BC cells

**Fig. 4:**
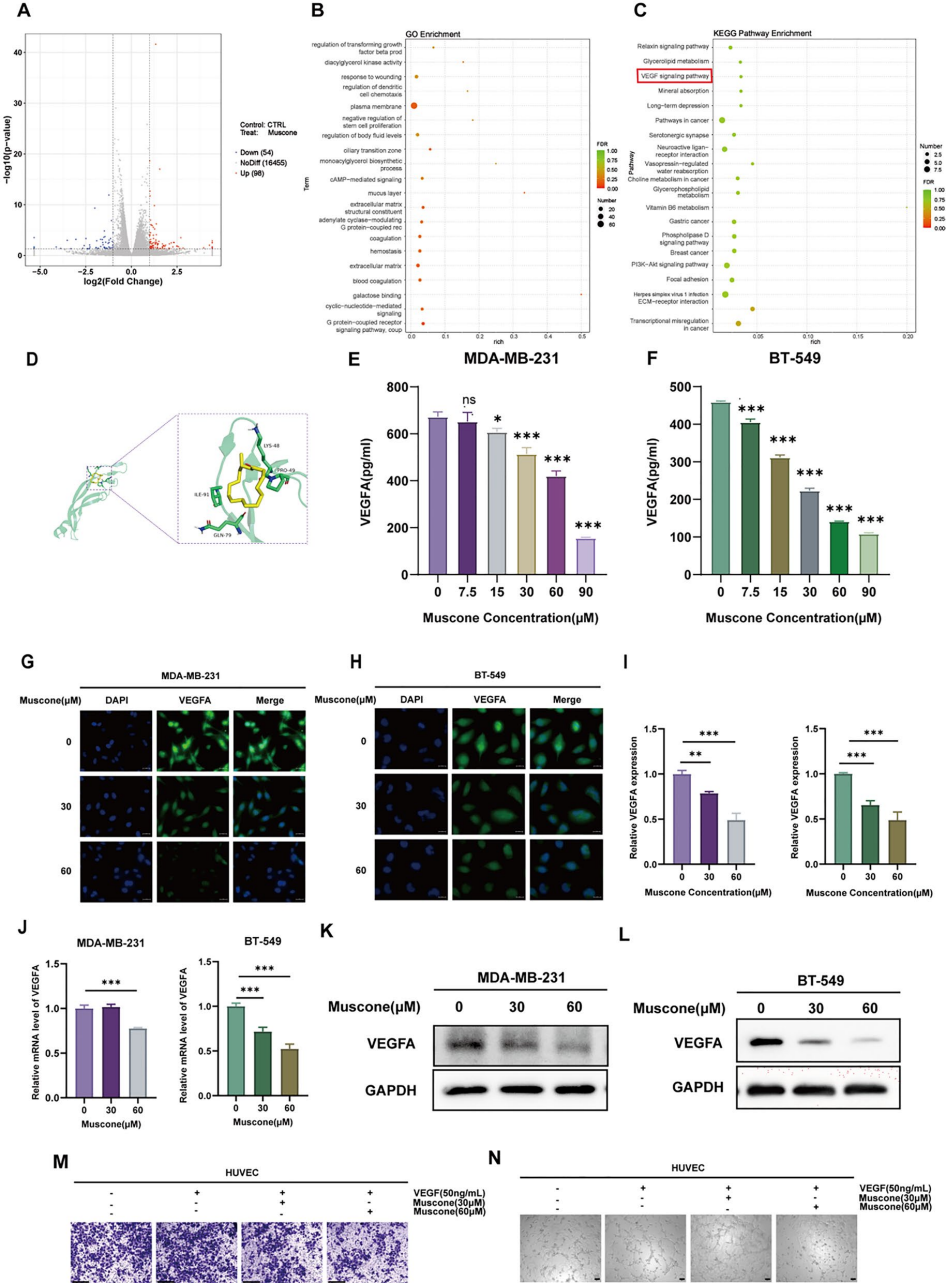
Muscone inhibits the VEGF axis in breast cancer (BC) cells. **A** Volcano plot of DEGs between control and muscone-treated MDA-MB-231 cells. Threshold was set at |log2FoldChange|> 1 and *P* < 0.05. Blue and red dots reflect diminished and elevated DEGs, respectively, by muscone treatment. **B** DEGs GO axis enrichment assessment. **C** DEGs KEGG network enrichment assessment. **D** Muscone interaction modes and locations with VEGFA, as estimated by AutoDock Vina software. **E**, **F** VEGFA secretion from BC cells was quantified by ELISA. **G**, **H** Cellular immunofluorescence (IF) assessments were conducted to assess VEGFA protein content in muscone-treated BC cells; Scale bar, 20 µm. **I**, **J** Western blot assessment of the muscone-mediated regulation of VEGFA protein levels in BC cells. **K**, **L** VEGFA transcript expression in BC cells as detected by RT-qPCR. **M** Muscone-mediated regulation of VEGF165-stimulated HUVECs migration as detected via Transwell cell migration assay; Scale bar, 1250 µm. **N** The muscone-mediated regulation of VEGF165-induced tube formation of HUVECs; Scale bar, 100 µm. Data shown as mean ± SEM. n = 3. *P < 0.05, **P < 0.01, and ***P < 0.001

To further examine the associated signaling network whereby muscone inhibits angiogenesis in BC, we performed transcriptome sequencing analyses on muscone-treated MDA-MB-231 cells. The DEGs between vehicle- and muscone-treated cells were identified according to two threshold values: fold-change (|log2FoldChange|> 1) and significance threshold (*P* < 0.05). In all, 152 genes were differentially regulated after muscone intervention, among which 98 were upregulated and 54 were downregulated (Fig. 4A). As shown in Fig. 4B, C, KEGG and GO assessment revealed that muscone-induced DEGs showed enrichment in the VEGF network, extracellular matrix constituent secretion pathways, and wound healing pathways. These findings corroborated with the above-mentioned evidences. Therefore, we speculated that the muscone-mediated tumor vasculature inhibition may be associated with the VEGF pathway.

As shown in Fig. 4D, molecular docking results suggested that the muscone molecule interacts with Gln79, Ile91, Lys48, and Pro49 of the VEGFA protein and formed a combination pocket, indicating that muscone might physically interact with VEGFA. In addition, muscone was shown to significantly reduce VEGFA secretion from MDA-MB-231 and BT-549 cells at 48 h post-treatment, based on ELISA assays of cellular supernatants (Fig. 4 E, F) and on IF analyses (Fig. 4G, H). It also lowered overall levels of VEGFA at the transcript (Fig. 4I, J) and protein (Fig. 4K, L) expressions in these two cell types.

To investigate whether muscone abrogates HUVECs cell migration and tube formation by suppressing VEGFA levels in TCM, we added recombinant VEGF165 instead of TCM to HUVECs. Here, Transwell cell migration assays and tube formation assays showed results consistent with TCM treated (Fig. 4M, N).

These results provided evidence that led us to us to conclude preliminarily that muscone alters angiogenesis in BC via interactions with the VEGF signaling pathway.

### Muscone affected VEGFR/PI3K/Akt/MAPK signaling in HUVECs

**Fig. 5:**
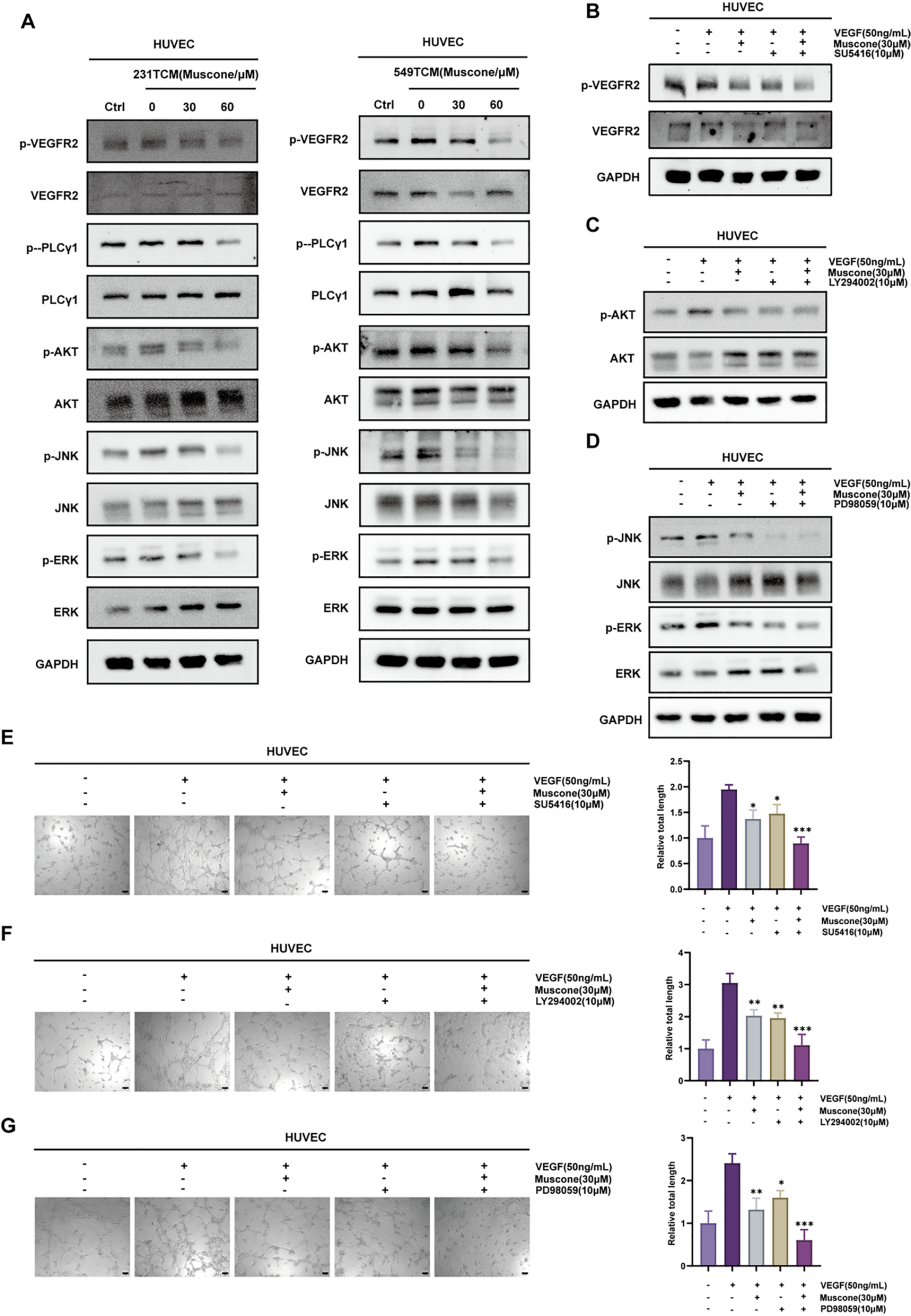
Muscone suppresses VEGFR2/PI3K/Akt/MAPK axis molecule activation in HUVECs. **A** Western blot assessment of VEGFR2, PLCγ1, Akt, JNK and ERK protein phosphorylation in HUVECs treated with TCM from muscone-pretreated MDA-MB-231 (231TCM) or BT-549 cells (549TCM). **B**–**D** Pharmacological inhibition of VEGFR signaling in HUVECs as determined by Western blot assessment. Cells were pre-exposed to the VEGFR2 inhibitor SU5416 (**B**), PI3K inhibitor LY294002 (**C**) or MAPK inhibitor PD98509 (**D**) for 1 h prior to stimulation with VEGF and muscone. **E**–**G** The effects of co-treatment with muscone and SU5416, LY294002, or PD98059 on VEGF-stimulated tube formation of HUVECs. The total tubular structure length was imaged and quantified via Fiji imaging software; Scale bar, 100 μm. Data provided as mean ± SEM. n = 3. *P < 0.05, **P < 0.01, and ***P < 0.001

VEGF family members of vascular endothelial developmental modulators regulate tumor angiogenesis and activate intracellular signaling pathways through binding to their receptors (VEGFR1-3), whereas the angiogenic response to VEGFA in vivo is mediated primarily through VEGFR2 activation [26]. Because VEGFR2 pathways are known to be the most essential angiogenic pathways that regulate endothelial cell functioning in angiogenesis, we used Western blot assays to explore whether muscone suppresses VEGFR2 and downstream protein phosphorylation. As shown in Fig. 5A, we found that VEGFR2 activation and stimulation of downstream networks, namely the PI3K/Akt and MAPK axes, induced by blank TCM were suppressed when treated with TCM from muscone-pretreated tumor cells. These experiments suggest that the decrease of secretion of VEGF from BC cells upon muscone treatment is sufficient to inhibit VEGFR signaling in downstream endothelial cells.

To confirm whether muscone regulates tumor angiogenesis in BC through VEGFR/PI3K/Akt/MAPK signaling, we tested our hypothesis by performing Western blotting and tube formation assays following treatment with a VEGFR2 inhibitor (SU5416), a MAPK inhibitor (PD98059), and a PI3K inhibitor (LY294002). As shown in Fig. 4B–D, the VEGFR2 phosphorylation status, ERK, JNK, and Akt were lower in cells co-exposed to muscone and either SU5416 (Fig. 4B), PD98059 (Fig. 4C), or LY294002 (Fig. 4D) when compared with proteins from cells treated with muscone alone. Tube lengths were also significantly reduced in these co-administered cells relative to cells exposed to muscone alone (Fig. 4E–G). Therefore, these findings corroborate with a model whereby muscone performs its antiangiogenic activity via VEGFR2/PI3K/Akt/MAPK axis inhibition in HUVECs.

### Muscone suppresses BC cell growth in vivo

**Fig. 6:**
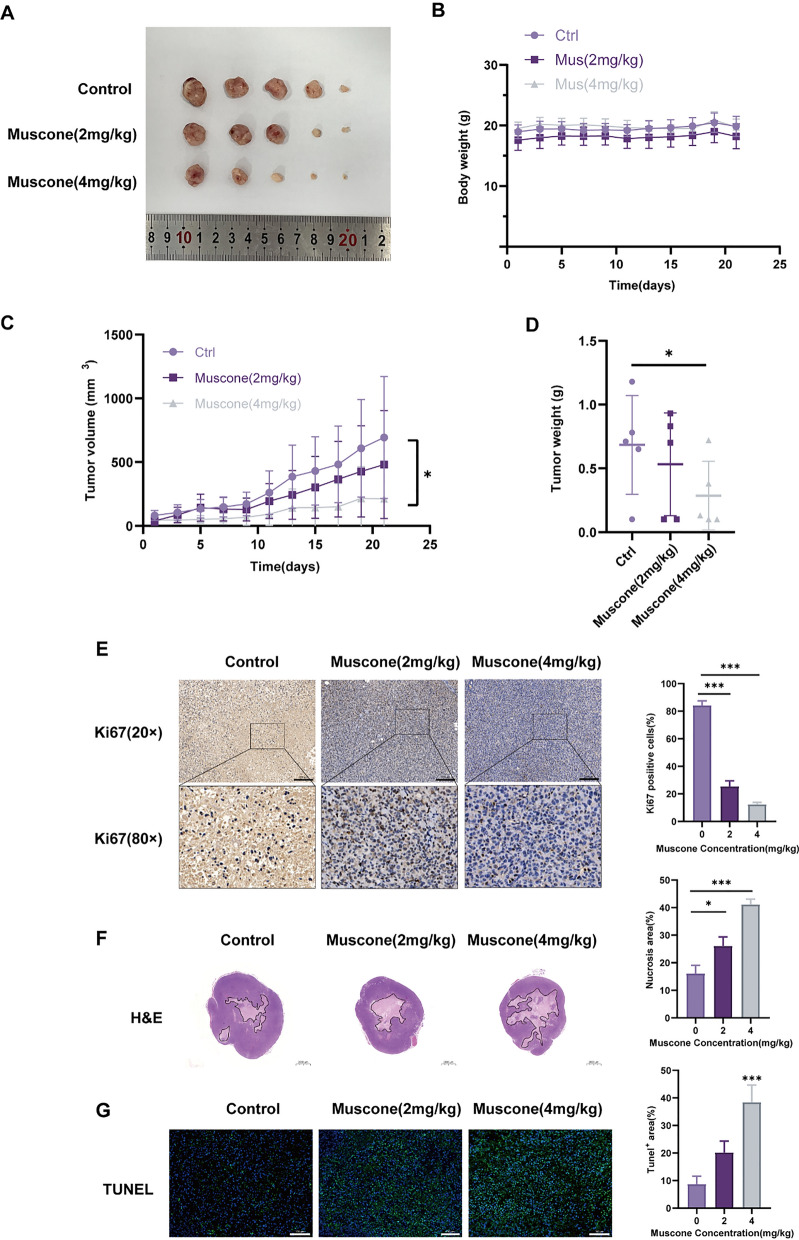
Muscone abolishes tumor development in a mouse breast cancer (BC) xenograft model. **A** Image of MDA-MB-231 tumors from different groups. **B** Body weights were recorded once every 2 days for 21 d. **C** Tumor volumes were computed once every 2 days. **D** Dissected tumor weights were accurately documented. **E** Representative immunohistochemical (IHC) Ki67 staining in MDA-MB-231 xenograft tumors; Scale bar, 200 µm. **F** Tumor tissue H&E staining; Scale bar, 2000 µm. **G** Tumor tissue TUNEL staining; Scale bar, 100 µm. Representative staining images and quantification. Data provided as mean ± SEM. n = 5 mice per group. *P < 0.05, **P < 0.01, and ***P < 0.001

To elucidate the muscone-mediated regulation of BC cell proliferation in vivo, MDA-MB-231 cells were subcutaneously administered into female BALB/c mice, which were then administered with muscone (2 or 4 mg/kg) or normal saline (Fig. 6A). As shown in Fig. 6B, no marked effect of muscone treatment on body weights was observed. However, consistent with our previous results, muscone treatment greatly suppressed in vivo tumor development in a dose-reliant fashion (Fig. 6C, D). Based on our IHC assessment, Ki67 content within tumors isolated from the muscone-treated mice was declined (Fig. 6E). Furthermore, muscone treatment produced a marked rise in the necrotic and apoptotic areas within tumors (Fig. 6F, G). These findings demonstrate that muscone suppresses BC cell proliferation in vivo.

### Muscone inhibits tumor angiogenesis and accelerates vascular normalization in vivo

**Fig. 7:**
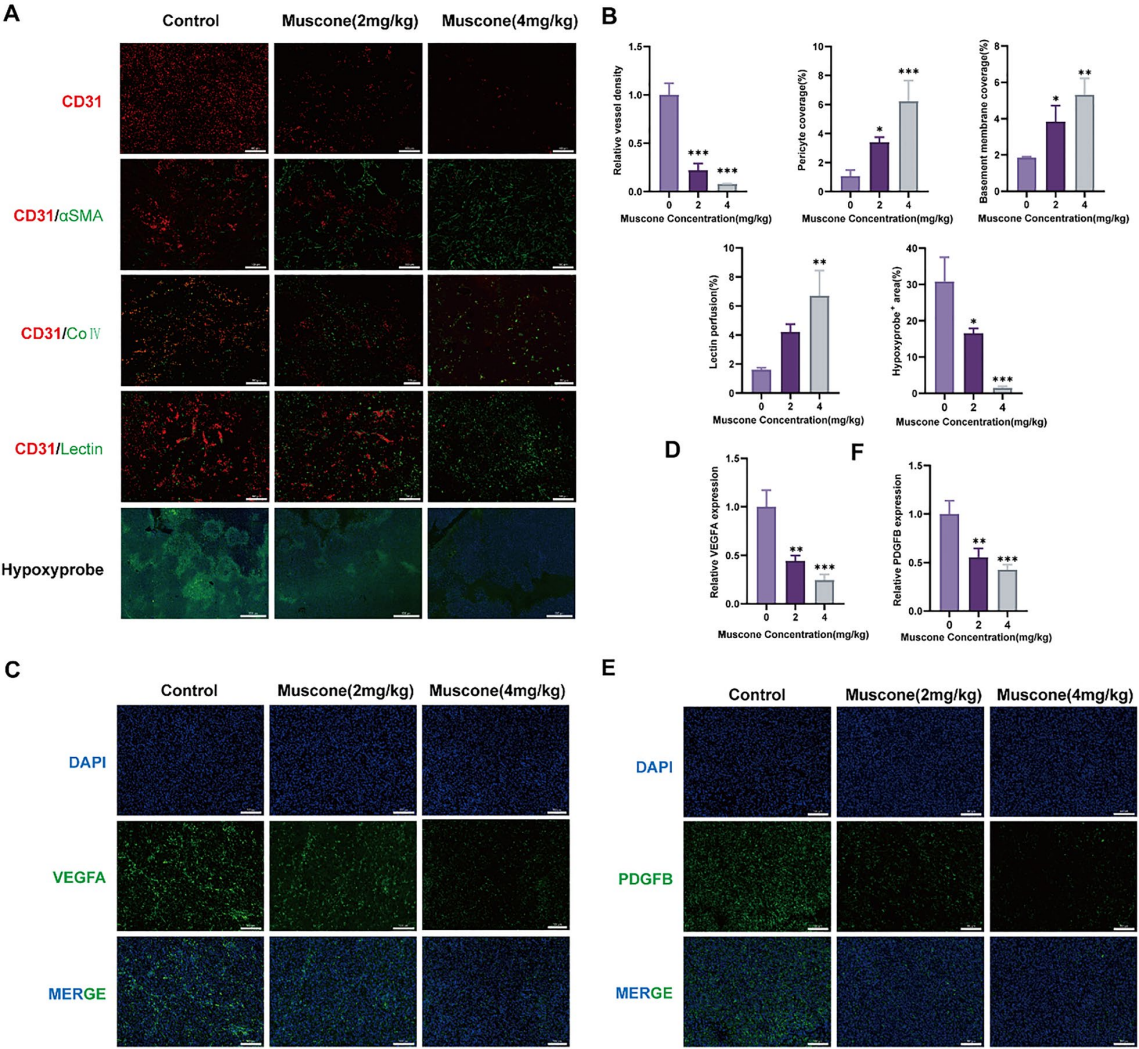
Muscone suppresses tumor angiogenesis. **A**, **B** Immunofluorescence (IF) images of tumor stained for CD31, αSMA, collagen IV (Col4), lectin, and hypoxyprobe in MDA-MB-231 xenograft tumors; Scale bar of CD31, αSMA, collagen IV (Col4) and lectin, 100 µm, scale bar of hypoxyprobe, 500 µm. Branching index, pericyte coverage, basement membrane coverage, lectin perfusion and hypoxic areas were recorded via Fiji, and quantitative data are provided as mean ± SEM. **C**–**F** IF pictures of VEGFA and PDGFB in MDA-MB-231 xenograft tumors; Scale bar, 100 μm. Representative staining images and quantification. Data provided as mean ± SEM. n = 5 mice per cohort. *P < 0.05, **P < 0.01, and ***P < 0.001

We further examined the significance of muscone in tumor angiogenesis in the BALB/c MDA-MB-231 xenograft model. When we characterized BC tumor vessels in mice treated with muscone, we found that the branching was reduced, relative to control mice, whereas, the α-smooth muscle actin (αSMA)-positive pericyte distribution along tumor vessels was elevated. Additionally, collagen IV, a critical endothelial cell basement membrane (BM) constituent, was augmented within tumor vessels of muscone-treated mice. The areas of the tumor that were marked by poor vessel perfusion and hypoxia were also significantly reduced in muscone-treated mice (Fig. 7A). Moreover, we found that the tumor tissue of muscone-treated mice expressed lower amounts of the proangiogenic factors VEGFA and PDGFB than did control mice (Fig. 7B, C), which potentially explained the rise in pericyte and BM coverage within tumor vessels of muscone-treated mice. Thus, we conclude that muscone treatment promoted tumor vascular normalization.

## Discussion

Angiogenesis, which delivers nutrients and oxygen to support sustained tumor growth, critically modulates solid tumor growth, and intense and rapid angiogenesis is characteristic of many malignant tumors [27]. In addition, angiogenesis also plays a key role in vascular remodeling, tissue injury, tumor cell migration and invasion [28–30]. Hence, suppressing tumor angiogenesis is a robust cancer prevention and intervention approach. In fact, multiple anti-angiogenic medications have been developed [31–33]. Most of these drugs are either monoclonal antibodies or small molecule tyrosine kinase inhibitors targeting VEGF or its receptors. Notably, monoclonal antibodies targeting VEGF are hindered from exerting an optimal therapeutic effect due to their high molecular weight and inability to penetrate solid tumors [34, 35]. Small-molecule tyrosine kinase inhibitors also lack notable anti-tumor effects due to their inability to inhibit all VEGF receptors [36–38]. Therefore, the establishment of more efficacious anti-angiogenic drugs, especially small-molecule compounds with low toxicity, is of critical importance.

Herein, we demonstrated that muscone inhibited BC cells (MDA-MB-231 and BT-549) proliferation and migration. It also strongly suppressed endothelial cell proliferation and migration that was induced by blank TCM, which is an important step in angiogenesis [39]. The antiangiogenic effect of muscone was also clearly observed in tube formation in cellular models*,* Matrigel plug angiogenesis assay in animal models and zebrafish angiogenesis assessment in vivo. Moreover, muscone treatment abrogated tumor development and promoted tumor vascular normalization in the BALB/c MDA-MB-231 xenograft model. Mechanistically, muscone inhibited BC angiogenesis by inhibiting the VEGFR/PI3K/Akt/MAPK axis. At the same time, we found that the anti-angiogenesis effect of muscone in BC cells (MDA-MB-231 and BT-549) was basically the same, indicating that muscone may have a broad anti-angiogenesis effect in breast cancer and is a candidate anti-BC angiogenesis drug.

MVD is an important biomarker of tumor angiogenesis, and evaluating tumor MVD is considered a robust method for antiangiogenic drug efficacy determination [30, 40]. Herein, we demonstrated that muscone strongly diminished CD31 content, which significantly lowered the corresponding in tumor tissue MVD values. Muscone also ameliorated tumor hypoxia and increased the coverage and perfusion of vascular wall cells. These findings confirmed the muscone anti-angiogenic activity in both cellular and animal models, suggesting that muscone is a robust therapeutic agent for suppressing tumor development and angiogenesis, and providing an theoretical foundation for further studies of muscone as an efficacious angiogenic suppressor for clinical applications.

VEGF was identified as a key DEG by RNA sequencing analysis in our studies. VEGF, through the binding and activation of VEGFR, can accelerate endothelial cell proliferation and migration, thereby inducing tumor angiogenesis and promoting tumor development [26, 41]. As the most important angiogenic factor known to date [42], VEGF has been implicated in multiple physiological and pathological events, such as, BC growth. Any drug that inhibits VEGF-associated events would be predicted to abrogate angiogenesis and thereby suppress tumor development and metastasis [39]. Herein, our finding that muscone suppressed VEGF levels in BC indicated that muscone inhibits angiogenesis by down-regulating VEGF content, and that this inhibition is potentially a mechanisms whereby muscone suppresses BC.

**Fig. 8:**
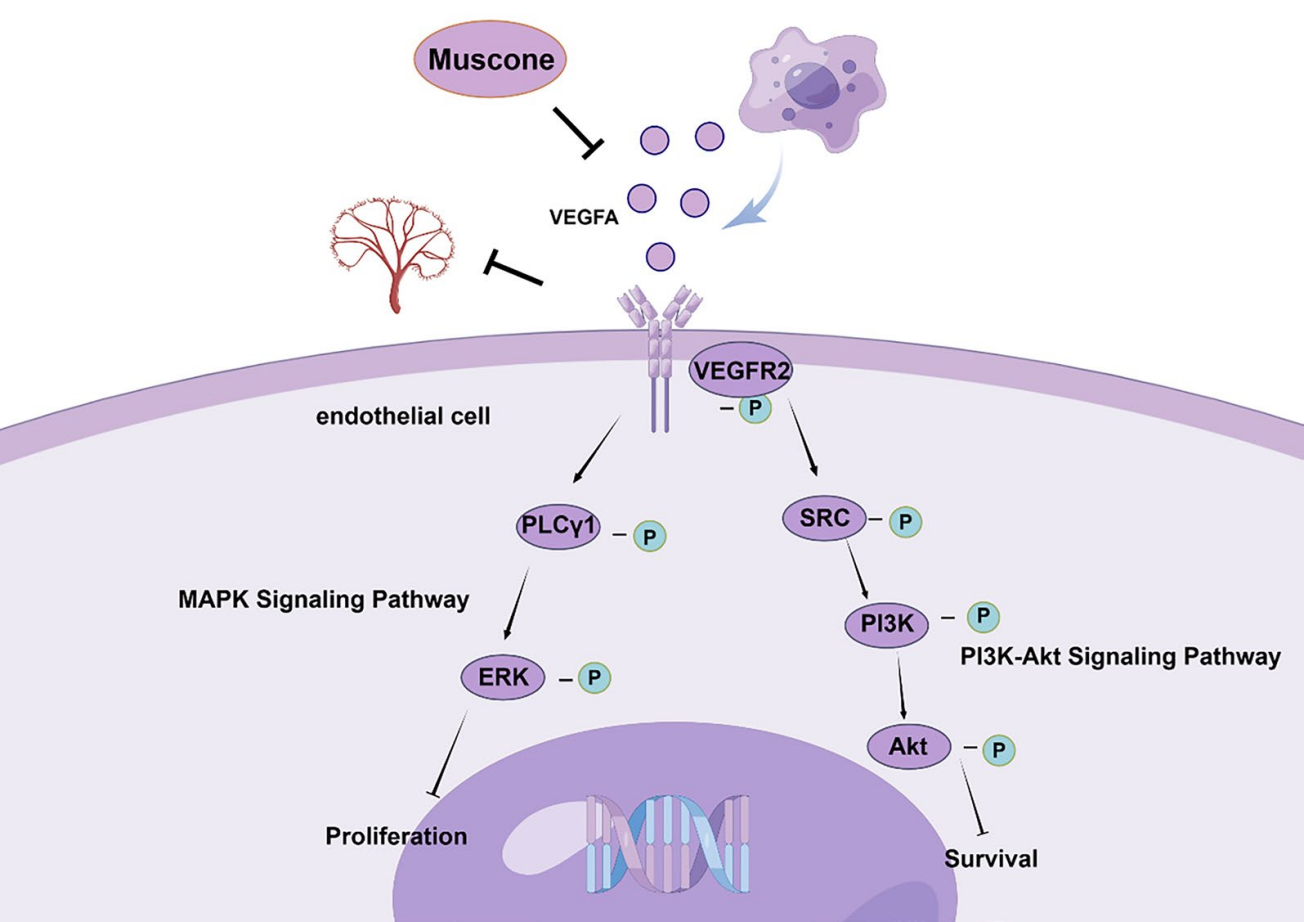
Schematic model illustrating the antiangiogenic mechanisms of muscone (by Figdraw)

VEGFR2 axis is a crucial network for tumor angiogenesis and includes the PI3K/Akt pathway and MAPK pathways. Phospholipase Cγ (PLCγ) is a critical regulator of VEGFR2-reliant proliferation [43–45]. PLCγ is stimulate by VEGFR2-based tyrosine phosphorylation, leading to hydrolysis of phosphatidylinositol 4,5 diphosphate (PIP_2_). PIP_2_ hydrolysis produces inositol 3,4,5 triphosphate (IP_3_) and Ca^2+^ fluxes as well as diacylglycerol, which subsequently stimulates protein kinase (PK) C activation, which then induces extracellular signal-regulated kinase (ERK) 1 and 2 activity and proliferation [44, 45]. PI3K is also a critical modulator of angiogenesis [46, 47]. VEGFA activates PI3K through multiple pathways, such as those involving FAK or Src [48, 49], or through direct binding of PI3K to a phosphorylated tyrosine (Tyr1175) in VEGFR2 [50]. PI3K is involved in endothelial cell tube formation as well as proliferation, survival, and tube permeability [51–53]. Akt, a PI3K signaling downstream intermediate, is of considerable significance in VEGF-based endothelial cell biology [53]. Akt is present in three isoforms (Akt1-3) in endothelial cells, and these enzymes are all PI3K activated via phosphatidylinositol-dependent kinase 1 (PDK1), the mammalian target of rapamycin C2 (mTORC2), and suppression of phosphatase PTEN [54]. Activated Akt is associated with angiogenesis through its effect on endothelial cell survival, proliferation, permeability, synthesis, and matrix metalloproteinases release [55, 56]. Here, Western blotting showed that muscone reduces TCM-stimulated VEGFR2 phosphorylation and its downstream network. This result suggested that muscone decreased secretion of VEGF from BC cells thus suppressed VEGFR2 activation and downstream proteins, which leads to the inhibition of BC angiogenesis, promoting tumor vascular normalization, and inhibiting BC progression. The proposed antiangiogenic molecular mechanisms of muscone are summarized in Fig. 8.

At present, chemotherapy is still the main treatment for BC. However, increasing evidence suggests that chemotherapy combined with anti-angiogenesis therapy and immunotherapy will further improve the efficacy, prolong RFS and improve the prognosis of patients [57, 58]. Compared with traditional antiangiogenic drugs, natural products have the advantage of less toxic side effects, although their effects are relatively mild. In future studies, we will further explore whether muscone can be used in combination with chemotherapy agents to improve antitumor effects on BC by enhancing chemotherapy efficacy and reducing side effects.

## Conclusion

Muscone was found to inhibit tumor angiogenesis via the VEGF/PI3K/Akt/MAPK network inhibition and to promote tumor vascular normalization, inhibiting BC progression. Based on our evidences, muscone is a promising natural anti-angiogenic intervention for BC management.

### Chemical compounds utilized in this research

Muscone (PubChem CID: 10947); SU5416 (PubChem CID: 5329098); LY294002 (PubChem CID: 3973); PD98059 (PubChem CID: 4713).

### Supplementary Information

Supplementary material 1.

## Data Availability

The data that support the findings of this study are available from the corresponding author upon reasonable request.

## Abbreviations

BC: Breast cancer
BRCA: Breast cancer susceptibility gene
CCK-8: Cell counting kit-8
DAPIL: 4′, 6-Diamidino-2-phenylindole
DEGs: Differentially expressed genes
DMSO: Dimethyl sulfoxide
EdU: 5-Ethynyl-2’-deoxyuridine
HUVECs: Human umbilical vascular endothelial cells
IC_50_: Half maximal inhibitory concentration
MVD: Microvessel density
ON: Overnight
PAGE: Polyacrylamide gel electrophoresis
PBS: Phosphate buffered saline
PS: Penicillin and streptomycin
PVDF: Polyvinylidene fluoride
TBST: Tris-buffered saline with 0.1% Tween-20
TCM: Tumor cell-conditioned medium
VEGF: Vascular endothelial growth factor
