# Supplementary material for: Muscone abrogates breast cancer progression through tumor angiogenic suppression via VEGF/PI3K/Akt/MAPK signaling pathways
Source: Cancer Cell Int. 2024 Jun 20;24:214. doi: 10.1186/s12935-024-03401-6 (PMC11188526; doi:10.1186/s12935-024-03401-6)
Supplement: Supplementary file 1 — Supplementary material 1. [file 12935_2024_3401_MOESM1_ESM.docx]

**Supplementary materials for**

**Muscone abrogates breast cancer progression through tumor angiogenic suppression via VEGF/PI3K/Akt/MAPK signaling pathways**

Danhong Wang^1,3,4*^, Xiaozhen Liu^3,4*^, Weimin Hong^2,3,4*^, Tianzheng Xiao^1,3,4^, Yadan Xu^2,3,4^, Xiang Fang^3,4,5^, Hongchao Tang^3,4^, Qinghui Zheng^3,4#^, Xuli Meng^3,4#^

^1^ College of Pharmacy, Zhejiang University of Technology, Hangzhou 310014, Zhejiang, China.

^2^ Zhejiang Provincial People's Hospital, Affiliated People's Hospital, Hangzhou Medical College, Hangzhou 310053, Zhejiang, China.

^3^ Department of Breast Surgery, General Surgery, Cancer Center, Zhejiang Provincial People’s Hospital , Affiliated People’s Hospital, Hangzhou Medical College, Hangzhou 310014, Zhejiang, China.

^4^ Key Laboratory for Diagnosis and Treatment of Upper Limb Edema and Stasis of Breast Cancer, Hangzhou 310014, Zhejiang, China.

^5^ College of Clinical Medicine, Jinzhou Medical University, Jinzhou 121001, Liaoning, China.

^*^ These authors contributed equally to this study.

^#^ Correspondence: Xuli Meng (mxlmail@126.com); Qinghui Zheng (zqh1530@126.com)

**Supplementary figures and figure legends**

**Figure S1.** MCF-10A cell viability when treated with muscone. Data are presented as the mean ±

SEM, n=3, **P* < 0.05, ***P* < 0.01 and ****P* < 0.001 when compared with control cells.

**
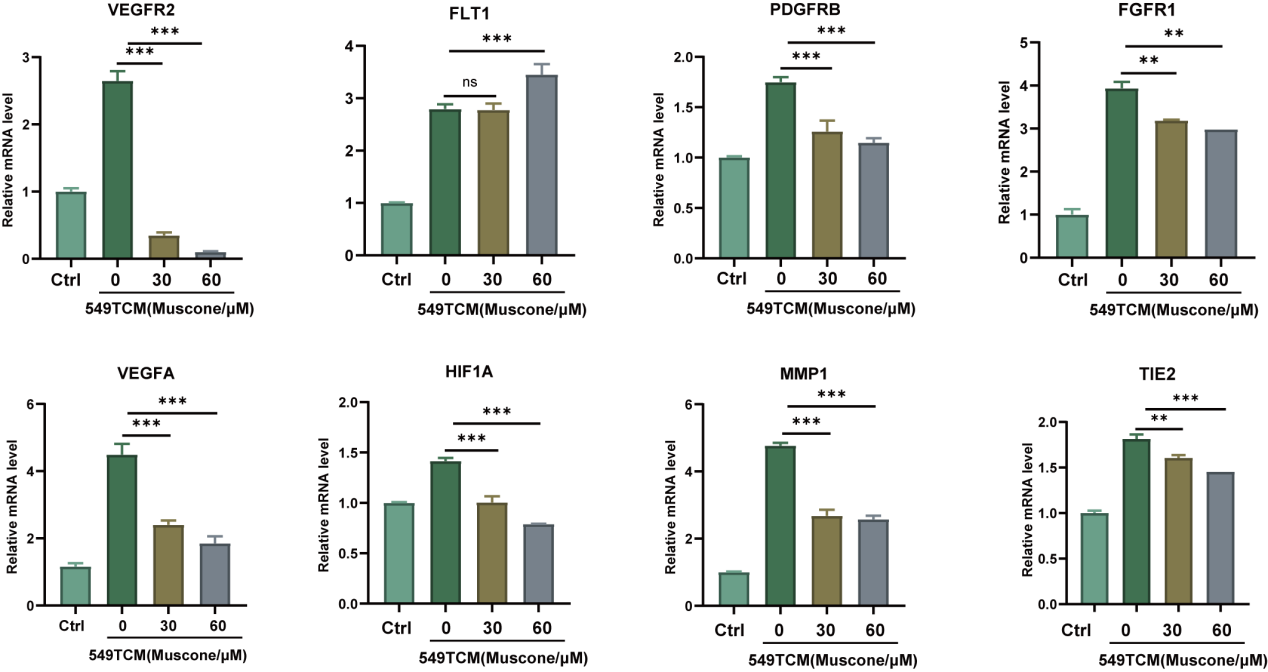
**

**Figure S2.** The effect of TCM from muscone-treated BT-549 cells (549TCM) on tumor angiogenesis-related gene expression in HUVECs. All data are shown as mean ± SEM, n = 3, **P* < 0.05, ***P* < 0.01, and ****P* < 0.001.

**Supplementary Tables**

**Table S1.** Primer sequences used for qPCR.

| Gene name | Forward (Sequence 5’ to 3’) | Reverse (Sequence 5’ to 3’) |
| --- | --- | --- |
| MMP1 | AAAATTACACGCCAGATTTGCC | GGTGTGACATTACTCCAGAGTTG |
| PDGFRB | AGACACGGGAGAATACTTTTGC | AGTTCCTCGGCATCATTAGGG |
| FGFR1 | CCCGTAGCTCCATATTGGACA | TTTGCCATTTTTCAACCAGCG |
| VEGFR2 | GGCCCAATAATCAGAGTGGCA | CCAGTGTCATTTCCGATCACTTT |
| FLT1 | TTTGCCTGAAATGGTGAGTAAGG | TGGTTTGCTTGAGCTGTGTTC |
| HIF1A | GAACGTCGAAAAGAAAAGTCTCG | CCTTATCAAGATGCGAACTCACA |
| TIE2 | TTAGCCAGCTTAGTTCTCTGTGG | AGCATCAGATACAAGAGGTAGGG |
| GAPDH | GTCTCCTCTGACTTCAACAGCG | ACCACCCTGTTGCTGTAGCCAA |

**Table S2.** Antibodies used in this study.

| Antibody | Catalog | Dilution | Company | Detection |
| --- | --- | --- | --- | --- |
| GAPDH | ab9485 | 1:3000 | Abcam | WB |
| Akt | #4685 | 1:1000 | Cell Signaling Technology | WB |
| p44/42 MAPK (ERK1/2) | #4695 | 1:1000 | Cell Signaling Technology | WB |
| Phospho-p44/42 MAPK (ERK1/2) (Thr202/Tyr204) | #4370 | 1:1000 | Cell Signaling Technology | WB |
| VEGF Receptor 2 | #2479 | 1:500 | Cell Signaling Technology | WB |
| JNK1+JNK2+JNK3 | ET1601-28 | 1:1000 | Huabio | WB |
| Phospho-JNK1/2/3 (JNK1(T183)+JNK2(T183)+JNK3(T221)) | ET1609-42 | 1:1000 | Huabio | WB |
| Phospho-Akt1 (Ser473) | ET1607-73 | 1:1000 | Huabio | WB |
| VEGFA | A0280 | 1:500 | ABclonal | WB/IF |
| Phospho-VEGF Receptor 2(Y1175 ) | AP0382 | 1:500 | ABclonal | WB |
| PLCγ1 | IPB5527 | 1:1000 | Baijia | WB |
| Phospho-PLCγ1 (Ser1248) | IPH0886 | 1:1000 | Baijia | WB |
| DAPI | C0060 | 1:500 | Solarbio | IF |
| PDGFB | AF02992 | 1:100 | Afantibody | IF |
| VEGFA | AF06392 | 1:200 | Afantibody | IF |
| CD31 | SAF005 | 1:100 | Afantibody | IF |
| αSMA | A2235 | 1:100 | ABclonal | IF |
| CoⅣ | AF11365 | 1:100 | Afantibody | IF |
| Lectin | DL-1177-1 |  | Vector labrototies | IF |
| Hypoxyprobe | HP1-100Kit |  | Biolead | IF |
| Ki67 | SAF008 | 1:100 | Afantibody | IHC |
| CY3 Goat Anti-Rabbit IgG | AFSA006 | 1:300 | Afantibody | IF |
| 488 Goat Anti-mouse IgG | AFSA002 | 1:200 | Afantibody | IF |
| Anti-Rabbit IgG H&L | ab6721 | 1:5000 | Abcam | WB |
